# Evaluation of a community-based intervention to improve maternal and neonatal health service coverage in the most rural and remote districts of Zambia

**DOI:** 10.1371/journal.pone.0190145

**Published:** 2018-01-16

**Authors:** Choolwe Jacobs, Charles Michelo, Mumbi Chola, Nicholas Oliphant, Hikabasa Halwiindi, Sitali Maswenyeho, Kumar Sridutt Baboo, Mosa Moshabela

**Affiliations:** 1 School of Nursing and Public Health, University of KwaZulu-Natal, Durban, South Africa; 2 Strategic Centre for Health Systems Metrics and Evaluations (SCHEME), School of Public Health, University of Zambia, Lusaka, Zambia; 3 Department of Epidemiology & Biostatistics, School of Public Health, University of Zambia, Lusaka, Zambia; 4 UNICEF, Lusaka, Zambia; 5 Department of Global Health, School of Public Health, University of Zambia, Lusaka, Zambia; 6 Africa Health Research Institute, Kwa-Zulu Natal, Durban, South Africa; Boston University School of Public Health, UNITED STATES

## Abstract

**Background:**

A community-based intervention comprising both men and women, known as Safe Motherhood Action Groups (SMAGs), was implemented in four of Zambia’s poorest and most remote districts to improve coverage of selected maternal and neonatal health interventions. This paper reports on outcomes in the coverage of maternal and neonatal care interventions, including antenatal care (ANC), skilled birth attendance (SBA) and postnatal care (PNC) in the study areas.

**Methodology:**

Three serial cross-sectional surveys were conducted between 2012 and 2015 among 1,652 mothers of children 0–5 months of age using a ‘before-and-after’ evaluation design with multi-stage sampling, combining probability proportional to size and simple random sampling. Logistic regression and chi-square test for trend were used to assess effect size and changes in measures of coverage for ANC, SBA and PNC during the intervention.

**Results:**

Mothers’ mean age and educational status were non-differentially comparable at all the three-time points. The odds of attending ANC at least four times (aOR 1.63; 95% CI 1.38–1.99) and SBA (aOR 1.72; 95% CI 1.38–1.99) were at least 60% higher at endline than baseline surveillance. A two-fold and four-fold increase in the odds of mothers receiving PNC from an appropriate skilled provider (aOR 2.13; 95% CI 1.62–2.79) and a SMAG (aOR 4.87; 95% CI 3.14–7.54), respectively, were observed at endline. Receiving birth preparedness messages from a SMAG during pregnancy (aOR 1.76; 95% CI, 1.20–2.19) and receiving ANC from a skilled provider (aOR 4.01; 95% CI, 2.88–5.75) were significant predictors for SBA at delivery and PNC.

**Conclusions:**

Strengthening community-based action groups in poor and remote districts through the support of mothers by SMAGs was associated with increased coverage of maternal and newborn health interventions, measured through ANC, SBA and PNC. In remote and marginalised settings, where the need is greatest, context-specific and innovative task-sharing strategies using community health volunteers can be effective in improving coverage of maternal and neonatal services and hold promise for better maternal and child survival in poorly-resourced parts of sub-Saharan Africa.

## Introduction

Over the past decade, considerable progress has been made in Zambia in reducing maternal and neonatal mortality. The Zambia Demographic Health Survey (ZDHS) estimates a 32% reduction in maternal mortality from 591 deaths per 100,000 live births in 2007 to 398 deaths per 100,000 live births in 2014 [1]. Despite this achievement, Zambia did not reach the targeted 75% reduction as per Millennium Development Goal (MDG) number five. Maternal and neonatal mortality have been associated with poor utilisation of essential maternal and neonatal health services such as antenatal care (ANC), skilled birth attendance (SBA) and postnatal care (PNC). Although utilisation of these services has improved in most developing countries [2], disparities in coverage of maternal health services have continued, with the most rural areas recording lowest coverage [3]. Similar to most developing countries [4, 5], deficiencies in the use of key maternal services in Zambia persist, with the poorest and most remote communities being least likely to benefit from these services [6]. In these communities, utilisation of key maternal health services is very low, much lower than the national estimates [6, 7]. For instance, in a recent study in selected remote areas, utilisation coverage for focused ANC, SBA and PNC within 48 hours were 30%, 37%, and 28% respectively [6], compared to 56%, 89%, and 81% for ANC, SBA and PNC respectively in urban areas [1].

Structural factors such as distance to the health facilities [8], cultural beliefs, income [9], mother’s livelihoods [6], and health system factors such as inadequate numbers and distribution of skilled health workers and poor quality of services [10], have been associated with poor coverage of maternal and neonatal health services in rural and remote areas. The human resources for health crisis in most developing countries, including Zambia, pose a significant challenge to the achievement of universal health coverage and the Sustainable Development Goals. Such challenges have led to increased attention to the potential of community-based interventions to expand access to essential health services, particularly in rural, remote communities [11].

Recent systematic reviews and other studies have demonstrated positive effects of community-based interventions on increasing access to and coverage of health services in rural areas [12–16]. In Zambia, the scope of existing literature largely covers interventions that aim to reduce child and neonatal mortality and to increase HIV services access and utilisation [17–19]. However, little is known about the effect of community-based interventions, such as Safe Motherhood Action Groups (SMAGs), on the uptake and utilization of maternal and neonatal health services, particularly in the most rural and remote areas as was done in Zambia. Safe Motherhood Action Groups (SMAGs) collectively represents community-based volunteer groups comprising various community health volunteers such as traditional birth attendants (TBAs), CHWs and neighbourhood health committee members. They also comprise both men and women trained in safe motherhood skills including identification of danger signs and creating awareness among rural women on the benefits of attending optimal maternal health services [20]. Although initially established in 2003 in Zambia, the programme was only implemented in rural and remote districts in 2012, and an evaluation component was, therefore, needed [20].

Through the Health for the Poorest Population (HPP) project, the Ministry of Community Development, Maternal and Child Health in Zambia, with support from UNICEF responded to the challenges of low coverage for maternal, neonatal and child health interventions among the poorest and most remote districts by training and supporting SMAGs in the four most rural districts where SMAGs were not implemented. The current study estimated the outcomes of this SMAGs intervention on coverage of maternal and neonatal health services, using ANC, SBA at delivery and PNC as interventions in the four most remote and poorest districts in Zambia.

### The Health for the Poorest Population (HPP) project

The HPP Project was designed to strengthen community-based services provided by community health workers (CHWs) by introducing SMAGs in the context of an equity-focused district health systems strengthening approach. The aim of the project was to improve access to and meet the national targets for coverage of quality maternal, neonatal and child health care services. The specific objectives of the HPP implementation strategy were to; increase the proportion of pregnant women attending at least four ANC visits to a minimum of 80%; increase the proportion of pregnant women receiving two doses of intermittent preventive treatment (IPTp2) (For prevention of malaria) during ANC to at least 80%; increase the proportion of skilled attendant deliveries to at least 60%; and increase the proportion of maternal and neonatal babies visited at home within 48 hours of birth to 20%.

The strategy used for improving access to and coverage of maternal and neonatal health (MNH) services had one main component of recruiting, training and providing support for volunteer SMAGs to provide essential MNH services in communities beyond a five-kilometre radius from a health facility. The main aim of SMAGs was to reduce critical delays in household-level decision-making about seeking life-saving maternal and neonatal care at health facilities, using mixed-groups of both men and women. SMAGs were specifically recruited and trained to function as health promoters and deliver essential information and create awareness about pregnancy, labour and neonatal health-related complications [20]. They were trained to; i) identify maternal and neonatal complications during pregnancy, delivery, and the PNC period; ii) refer women with maternal and neonatal problems for management at health facilities; iii) encourage pregnant women to go for regular ANC visits, delivery, and PNC in a health facility; and iv) to provide birth preparedness messages to pregnant women and their spouses in the community about seeking life-saving maternal and neonatal care at health facilities [20]. A standard training programme of six weeks was used to empower SMAGs with knowledge and skills specifically for promoting antenatal care, delivery in a health facility with a trained provider, postnatal home visits, and essential neonatal care.

The implementation of the intervention began in 2013 for a two year period. Before the implementation of this community–based intervention, a baseline household survey was conducted in 2012 to determine the baseline population level coverage of the key MNH intervention. Monitoring and evaluation of the intervention was conducted through quarterly review meetings, planned field visits, and supportive supervision. The outcome evaluation of the HPP project was conducted through repeat household surveys at midline in 2013 and endline in 2015, the results of which are the focus of this study.

## Methods

### Study setting

This study was conducted in Mungwi, Luwingu, Samfya and Chiengi Districts, located in Luapula and the northern provinces of Zambia. These districts were purposively sampled as being the poorest among the thirteen poorest districts, where the most vulnerable and marginalised people live in Zambia. The population distribution of vulnerability, poverty and deprivation was based on national data analysis [21, 22]. In addition, the sampled districts had the highest maternal and child mortality rates [1].

### Study design

The study used a “before—and—after” multi-stage evaluation approach with repeat cross-sectional household surveys at baseline, midline and endline between 2012 and 2015. The baseline survey was conducted in 2012, the midline in 2013 and the endline in 2015. The Lot Quality Assurance Sampling (LQAS) method was used, originally considered as a quality assurance approach, but has been adapted to evaluate public health interventions and services, especially in developing countries [23–25]. The small sample design of LQAS is based on the binomial distribution [26]. Prior studies suggest that LQAS is an efficient sampling design used when one wants to identify general program coverage or indeed communities having inadequate service coverage [27–29]. Therefore, in the context of the poorest and most remote districts of Zambia, LQAS was considered a suitable sampling design for evaluating the intervention because of the small sample size it requires for each cluster.

### Sampling strategy

Each district was subdivided into 5–9 administrative sub-district strata called Supervision Areas or catchment area with a dedicated health facility responsible for delivering health services [6]. From the four selected districts, a total of 29 SAs, nine from both Chiengi and Samfya, six from Luwingu, and five from Mungwi were included in the study. Using the WHO LQAS guide, a list of all the villages in each supervision area, used as a sampling frame, was retrieved from the 2010 population census, and the probability proportional to size technique was followed to select 19 villages from each supervision area [27]. Probability proportional to size (PPS) ensures that sample villages are selected based upon their proportional representation of the entire population [6, 30]. When aggregated at the district level, the sample size of 19 for each supervision area provides district-level coverage proportions for key indicators with a 95% confidence interval not exceeding 10% [24].

The segmentation sampling approach, advocated in survey guidelines was used as more rigorous second-stage sampling technique [29, 30]. Once a reference house was selected, the next closest house was selected for an interview. This additional technique was used to reduce the chance of a house having a zero probability of selection [30]. Individual respondents at household level were randomly-selected from the PPS-selected villages using a random sampling technique.

The main inclusion criterion for households was the presence of mothers with children aged 0–5 months and mothers that lived in the households during pregnancy and delivered their baby within the same study area. In instances where two or more eligible mothers were found in the same household, simple random sampling was done to select one respondent. The sampling frame and design was the same across the three surveys and has been described in previous studies [6, 29, 31]. The total sample size of study participants for baseline (551), midline (550) and endline (551) surveys combined was 1652 women.

### Data collection

At baseline, a pre-tested structured interviewer-administered paper questionnaire, designed in the local language of Bemba, was used to collect data from participating mothers. At midline and endline, the same structured questionnaire was used on Magpi's mobile data platform, which uses mobile phones for data collection and a real-time cloud-based system for data storage [32]. Trained research assistants conducted the interviews, with questions on demographic characteristics and various health-related behaviours about utilisation of antenatal care services, facility delivery services, and PNC services, and these questions were repeated at all three-time points.

### Outcome variables

The evaluation was based on eight core outcome indicators: attending ANC at least once during pregnancy; receiving ANC once from a skilled birth attendant; attendance of ANC at least four times during pregnancy, receiving at least two doses of intermittent preventive treatment (IPTp2) during pregnancy; SBA at delivery; SBA at a health facility; PNC within 48 hours after birth from an appropriate provider; and PNC within 48 hours from a SMAG. The variable, SBA was generated, to include; nurse, midwife and doctor who undergo obstetrical training.

### Explanatory variables

The surveys collected data on mother’s age, level of education, literacy level, marital status, HIV test results and receiving IPTp2 during pregnancy. Additional data collected at midline and endline included distance to the health facility, receiving messages on birth preparedness from a SMAG or a CHW, receiving messages on birth preparedness from other providers and presence of a trained SMAG within the community.

### Data management and analysis

Data were cleaned and appended to create a new dataset containing the baseline, midline and endline data, with a total of 1652 participants. The ‘svy set’ syntax in Stata version 13 [33] was used to adjust estimates to account for the complex multistage sampling design and the clustered nature of the data. Weighted proportions were calculated to adjust for disproportionate sampling of mothers’ use of MNH services for each category in the explanatory variables. The trends in coverage over time were also calculated using a Mantel-Haenszel chi-square test for trends to detect differences between time points. To assess the outcome of the intervention on each dependent variable, logistic regression models were used to obtain odds ratios (ORs) and 95% confidence intervals (CIs). The ORs were adjusted for individual and community-level characteristics. The baseline time point was used as the reference category in all analyses. For data not collected at baseline, the midline point was used as reference category. Predictors that were significant at p<0.2 in univariate analyses were included in multivariate model. All statistical analysis was conducted using Stata 13.

### Ethical considerations

This study was approved by the Tropical Disease Research Centre (TDRC; Ref No: TRC/C4/07/2015) in Zambia and the University of KwaZulu-Natal (Ref No: BE363/15) in South Africa. Formal permission was also obtained from the Ministry of Community Development, Mother and Child, and from the traditional leaders through the District Medical Officers (DMOs) of the respective selected districts. Informed written or thumbed print consent was obtained from each study participant after explaining the objectives of the study and procedure.

## Results

### Participant characteristics

Overall, 1652 (551 at baseline, 550 at midline and 551 at endline) mothers were interviewed and included in the analysis. As shown in Table 1, mothers surveyed at all three-time points had similar demographic characteristics. Over half (60% at baseline, 57% at midline and 52% at endline) of mothers surveyed were aged 25 years and above. Although over three quarters (80% at baseline, 78% at midline and 83% at endline) of mothers reported having attended school, over two-thirds could not read at all and this factor did not vary by survey. The midline and endline samples had a much higher percentage (above 80%) of married mothers. Half of the mothers interviewed both at midline and at endline reported living beyond 5km from the nearest health center.

**Table 1:** Characteristics of mothers at Baseline, Midline and Endline surveys for the intervention.

Variable	Baseline	Midline	Endline	*p* value
	(n = 551)	(n = 550)	(n = 551)	
	%(95% CI)	%(95% CI)	%(95% CI)	
**Individual Characteristics**				
**Age**				
15–24	40 (36–46)	41 (36–45)	43 (38–47)	0.517
25 and above	60 (56–64)	59 (55–63)	57 (52–61)	
**Marital Status**				
Not married	35 (32–38)	11 (9–14)	13 (10–16)	0.000
Married	65 (62–68)	89 (86–91)	87 (84–90)	
**Ever attended school**				
No	20 (16–23)	22 (18–25)	17(13–19)	0.117
Yes	80 (77–84)	78 (74–81)	83 (81–87)	
**Level of education**				
Never been to school	21(17–24)	25 (21–29)	20 (17–24)	
Incomplete primary	53 (48–57)	42 (38–46)	42 (38–47)	
Complete primary	16 (13–19)	14(12–18)	15 (12–18)	
Post Primary*	10 (8–13)	19 (16–22)	21 (18–25)	0.000
**Women’s Literacy**				
Cannot read at all	63 (58–68)	63 (58–67)	59 (55–63)	
Can read**	37 (33–41)	37 (33–41)	41 (37–45)	0.244
**Received Messages on birth preparedness**				
No	##	16 (13–19)	10 (7–12)	
Yes	##	84 (81–87)	90 (87–93)	0.000
**Received Messages on birth preparedness from SMAG/CHW**				
No	##	49 (37–47)	31 (27–35)	
Yes	##	51 (53 - .62)	69 (64–73)	0.000
**Community Charactistics**				
**Presence of a trained SMAG/CHW within the community**				
**No**	##	53 (48–58)	30 (25–36)	
Yes	##	47 (43–51)	70 (66–74)	0.000
**Distance to the health facility**				
Within 5 Km	##	47 (43–52)	48 (43–52)	
Beyond 5 km	##	53 (48–57)	52 (48–56)	0.975

* Post primary means attempted and completed secondary

## Variable not measured at baseline

**Can read included those that could read part of the sentence; F test accounting for complex sampling design

#### Antenatal care indicators

**Fig 1:**
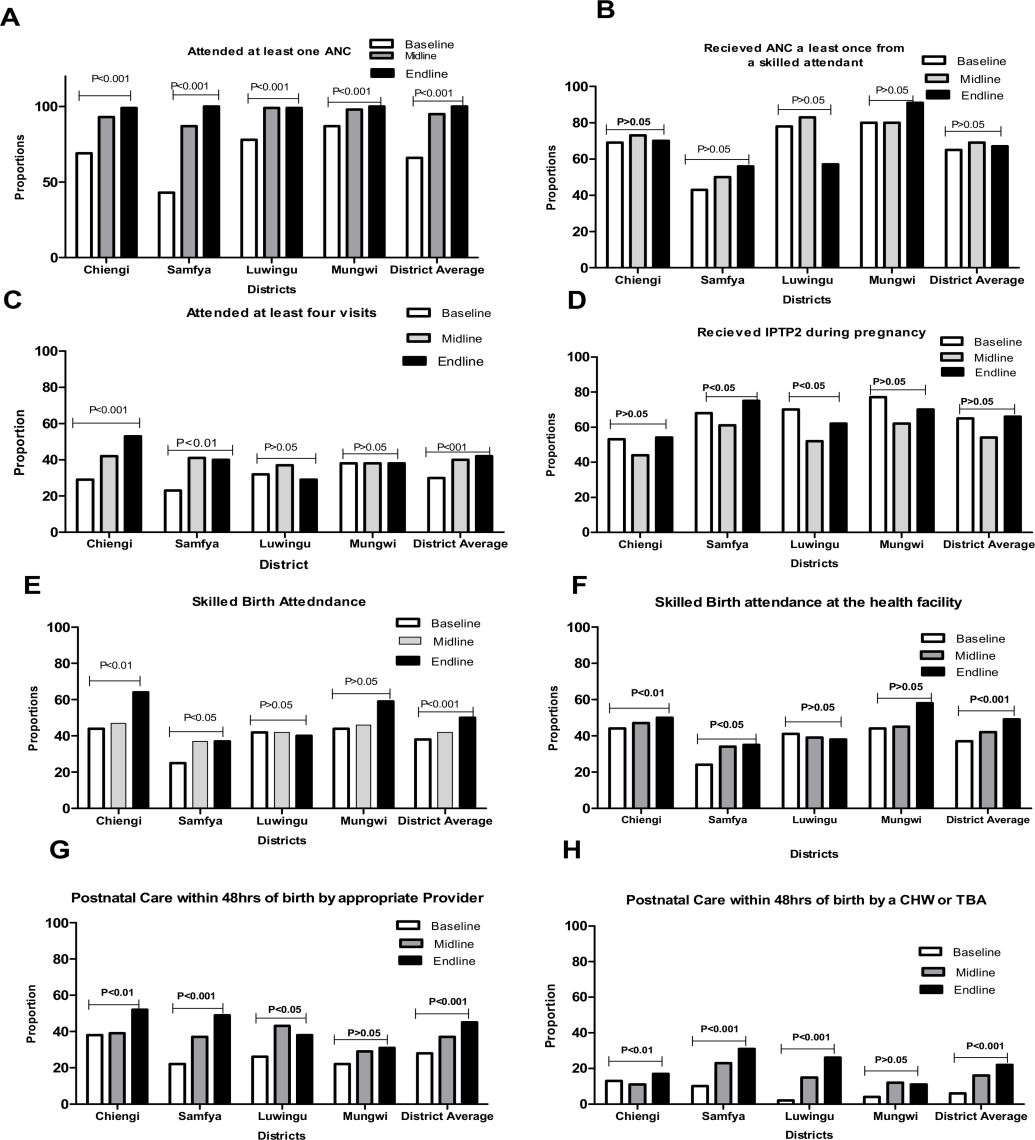
Trends and differentials in the coverage for ANC, SBA and PNC indicators over a three year period.

Fig 1 presents differentials and trends in the coverage of core indicators for MNH services shown for each district. Fig 1A, 1B, 1C and 1D show that, over time, there was a significant increase (*p*<0.01) in the mean weighted coverage for ANC at least once and ANC at least four times. However, there was no significant increase in the coverage of ANC at least once from a skilled provider. At the district level, no significant change in coverage was observed between baseline and endline for ANC at least once from a skilled provider in Luwingu (*p*>0.05) and Mungwi (*p*>0.05) as shown in Fig 1C. The average district coverage for mothers that received IPTp2 during their last pregnancy did not change between baseline (65%) and endline (66%), but did decline in the interim period between baseline and midline from 65% to 54% before rebounding at endline (Fig 1D). As shown in Table 1, the proportion of mothers that reported having a trained SMAG in their community increased significantly (*p<*0.001) from 47% at midline to 70% at endline. Further, the proportion of mothers that received birth preparedness messages from a SMAG also increased significantly (*p* <0.001) by 18% between midline and endline surveys.

#### Skilled birth attendance

A steady significant increase (*p*<0.01) in the weighted mean proportion of women being attended by a SBA was observed. As shown in Fig 1E, 50% of women in the four districts had skilled birth attendance during delivery at endline compared to 40% at baseline. The patterns of increase for this indicator were also observed at the district level, except for Luwingu and Mungwi where a decrease in coverage was observed for both indicators.

#### Postnatal care indicators

The mean weighted proportion of mothers seen by an appropriate provider within 48 hours after birth increased significantly (*p*<0.001) from 28% at baseline to 37% at midline and 45% at endline. The mean proportion for mothers seen by a SMAG within 48 hours after delivery also significantly increased from 15% at midline to 22% at endline (P<0.001). However, in Mungwi district, no significant increase in coverage for mothers that received PNC within 48hours after delivery from a SMAG (CHW or TBA) was observed, as shown in Fig 1H.

Table 2 presents the outcome of the community-based intervention over time in the four selected districts after adjusting for socio-demographic factors. The odds of attending ANC at least four times were 63% higher at endline than at baseline (aOR 1.63; 95% CI 1.38–1.99). At endline, mothers were 1.72 times more likely to have skilled birth attendance at delivery compared to baseline (aOR 1.72; 95% CI 1.35–2.10). Further, a two-fold increase in the odds of PNC by an appropriate provider by mothers surveyed at endline was observed compared to mothers surveyed at baseline (aOR 2.13; 95% CI 1.62–2.79). Table 2 also shows a linear increase in coverage of PNC received from a SMAG within 48 hours after delivery was observed from midline (15%) to endline (22%). However, after accounting for socio-demographic factors, there was no significant change (P>0.05) in the likelihood of mothers receiving ANC at least one from a skilled attendant, and receiving at least two doses of Intermittent preventive treatment in pregnancy.

**Table 2:** Logistic regression results of changes in the core maternal health care outcome indicators in four selected remote districts of Zambia after the community-based intervention (2012–2015).

Indicator	Coverage%(n/N*)	**Adjusted OR (95%CI)	P value for trend
**Women that received one ANC by skilled attendant**			
Baseline	64% (356)	1	0.416
Midline	69% (381)	1.20 (0.92–1.58)	
Endline	66% (367)	1.06 (0.81–1.37)	
**Women that had at least Four ANC visits**			
Baseline	29% (165)	1	<0.001
Midline	40% (220)	1.44 (1.11–1.87)	
Endline	42% (233)	1.63 (1.38–1.99)	
**Received IPT2 during Pregnancy**			
Baseline	65% (360)	1	0.973
Midline	54% (297	0.60 (0.48–0.80)	
Endline	66% (362)	0.98 (0.75–1.21)	
**Skilled Birth Attendant**			
Baseline	37% (209)	1	<0.001
Midline	43% (234)	1.25 (0.97–1.62)	
Endline	49% (270)	1.72 (1.35–2.10)	
**Postnatal care within 48 hours by an appropriate provider**			
Baseline	28% (154)	1	<0.001
Midline	37% (206)	1.61 (1.26	
Endline	45% (245)	2.13 (1.62	
**Postnatal care (neonate) within 48 hours SMAG (CHW/TBA)**			
Baseline	#	#	<0.001
Midline	15% (72)	1	
Endline	22% (99)	1.58 (1.14–2.10)	

Response rate was 100%

Community based interventions were targeted at training SMAGs who included CHW and TBAs on maternal health skills

*The total study participants was 551 at baseline, 550 at midline and 551 at endline

**Adjusted for woman’s age, marital status, ever attended school, women’s literacy, and distance to the facility and level of education

# Baseline data for SMAGs was not collected as these were not yet trained

The results in Table 3 present a summary of the multivariate models for each of the three selected core indicators of maternal health services to assess predictors of the use of the services at endline. Distance to the health facility was a significant predictor of ANC utilisation at least four times (aOR 0.58 95% CI 0.45–0.75) with mothers that reported living beyond 5km of the nearest health facility being less likely to utilise ANC at least four times compared to those that reported living within 5km of the nearest health facility. Distance to the nearest health facility was also a predictor of SBA (aOR 0.62 95% CI 0.47–0.81) with mothers that reported living beyond 5km to the nearest health facility less likely to have had SBA compared to those living within 5km of the nearest health facility. Mothers who could read had a higher likelihood of SBA (aOR 1.44 95% CI, 1.14–1.99) compared to those that could not read. Receiving birth preparedness messages from a SMAG or CHW and receiving HIV test results during the most recent pregnancy were significantly associated with SBA. Furthermore, mothers who received ANC at least once from a skilled provider had a four-fold likelihood of SBA (aOR 4.01 95% CI, 2.88–5.75), and a two-fold likelihood of receiving PNC within 48 hours by an appropriate provider (aOR 2.11 95% CI, 1.49–3. 68). Mothers that had a SBA at delivery were twice as likely to receive PNC within 48 hours after birth (aOR 2.41 95% CI, 1.82–3.15). Mothers who had SMAGs within the community were twice as likely to receive PNC at endline compared to mothers who did not (aOR 1.86 95% CI, 1.26–2.73).

**Table 3:** Results of the logistic regression analysis of the factors related to the endline outcome of use of postnatal care by a CHW or SMAG provider over a three year period.

Variables	Proportions N = 551 % (n)**	ANC at least 4 times		Skilled Birth Attendance		postnatal care	
		Adjusted Odds Ratio (95% CI)	P-value	Adjusted Odds Ratio (95% CI)	P-value	Adjusted Odds Ratio (95% CI)	P-value
**Mother’s Literacy**							
Cannot read at all		1		1		1	
Can read*		1.40 (0.96–2.03)	0.081	1.44 (1.14–1.99)	0.028	1.59 (1.17–2.66)	0.030
**Distance to the health facility**							
Within 5 Km	48 (262)	1		1		1	
Beyond 5 km	52 (289)	0.58 (0.45–0.75)	0.000	0.62 (0.47–0.81)	0.000	1.03 (0 .79–1.35)	0.126
**Received HIV test Results during Pregnancy**							
No	15 (84)			1		1	
Yes	85 (465)	#	#	1.72 (1.13–2.61)	0.012	2.04 (0.95–4.37)	0.068
**Presence of a trained SMAG within the community**							
No		1		1		1	
Yes		1.20 (0.93–1.56)	0.160	1.29 (0.96–1.72)	0.096	1.86 (1.26–2.73)	0.002
**‘Received Messages on birth preparedness from SMAG**							
No	31 (158)	1		1		1	
Yes	69 (349)	1.70 (0.97–2.99)	0.065	1.49 (1.10–2.00)	0.009	1.76 (1.20–2.19)	0.004
**Received one ANC visit provided by skilled provider**							
No	33 (181)			1		1	
Yes	67 (366)	#		4.01 (2.88–5.72)	0.000	2.11 (1.49–3.68)	0.008
**Skilled Birth Attendance**							
No	50 (276)					1	
Yes	50 (273)	#		#		2.41 (1.82–3.15)	<0.001

# Predictor variable not included in the model for the individual outcome variable

*Women that could read the whole sentence or part of the sentence;

**Coverage at endline

## Discussion

Our findings suggest that training and support for SMAGs, community-based action groups comprise and target both men and women in the strengthening of MNH services was associated with increased coverage of key indicators of MNH interventions among women in the selected poor and very remote districts. We found a significant increase in ANC attendance at least four times, SBA at delivery and receipt of PNC within 48 hours after delivery. These findings are consistent with studies that have evaluated the role of women’s groups in improving MNH services [31, 34]. On the contrary, the coverage of ANC once from a skilled provider and receipt of IPTp2 during pregnancy, both considered as proxies for quality care did not improve at endline. This study further showed that receiving birth preparedness messages from a SMAG, receiving HIV test results during the most recent pregnancy and receiving ANC at least once from a skilled provider were positive predictors for use of SBA at delivery and PNC within 48 hours after deliver.

Although national targets for most of MNH indicators, receipt of ANC at least four times and SBA at delivery 80% and 60% respectively, were not met by endline following the intervention, the observed significant increase in the coverage for these core indicators holds promise for these marginalised and remote communities of Zambia. Similar findings were reported in rural Ethiopia where a community sensitization intervention revealed an increase in the coverage for most MNH indicators [31]. Findings from similar settings [31], as in this study, also show that training CHWs in safe motherhood strategies that target marginalized populations is critical for addressing poor utilisation of MNH services [31, 35–38].

Further, the association between receiving birth preparedness messages from SMAGs and receiving SBA at delivery and PNC within 48 hours by women suggests the essential role that SMAGs can play as champions of community health systems and health promotion platforms for safe motherhood [39]. However, it is also possible that the messages by SMAGs were sent to women who could have been most likely to deliver in a facility. Nevertheless, Prost et al. [13] argues that using community engagement strategies such as community interventions (such as SMAGs) within existing health system for maternal and newborn health could positively influence demand for MNH health care. Therefore, with increasing attention being paid by most developing countries towards strengthening health systems and community engagement, enhancing community-based services and creating strong links between communities and health facilities through SMAGs has the potential for improving coverage of MNH interventions in the poorest, most remote districts and communities [39].

Efforts to improve utilisation of PNC within 48 hours after birth by an appropriate provider has remained a challenge in rural areas of Zambia [1], and worse in remote areas [6], yet most maternal deaths occur during this period. Conversely, a significant two-fold and four-fold increase in women’s likelihood to receive PNC within 48hrs by an appropriate provider and a SMAG respectively points to the potential of this equity-focused intervention approach for increasing coverage of PNC services. We are aware that a number of studies in developing countries have reported cultural factors such as seclusion of neonates indoor for over a month as barriers to utilisation of PNC services [40]. The experience in this Zambian study has shown that expanding access to PNC and enhancing community engagement through the training and support of community health volunteers such as SMAGs can overcome these and other barriers faced by mothers particularly in poor and remote communities and provide hope for neonatal and maternal survival.

Furthermore, a lack of significant change at endline for women’s receipt of IPTp2 during pregnancy, a proxy for quality of ANC, suggests need for further research to explore the feasibility of task shifting some roles, including distribution of IPTp for malaria prevention, away from doctors and nurses to the SMAGs and/or CHWs as a means to improve access to basic MNH services [38].

Some demographic and geographical contexts such as mothers’ literacy levels and distance to the health facilities remain critical and could explain failure of the intervention to meet the national targets [41–43]. Consistent with findings in previous research [8, 44–46], distance to the nearest health facility remains a challenge for access and utilisation of health services even with community-based approaches. All these perspectives are viewed as crucial in improving utilisation of MNH services. Strategies that address access factors (distance) within community-based interventions need to be considered to enable mothers reach the health facilities. We also recommend that literacy programmes in remote areas must be enhanced among mothers and girl children.

This study had some limitations worth mentioning. Firstly, the uncontrolled before-and-after design used in this study did not allow us to get a control group to compare outcomes of the intervention, hence there could have been some threats to internal validity. Secondly, other routine district events, such as mobility of personnel or other maternal health care related programmes, which were not part of the intervention, could have affected the outcomes between the “before” and “after” measurements. Further, facility-based data which may have explained some effects on the outcomes was not collected at midline and endline and hence was not included in the analysis. Thirdly, social desirability is another possible limitation. Participants may have given responses that were thought to be the best desired practices. However, although these limitations could have occurred, we think they do not significantly influence our findings because; firstly, the evaluation was done at three-time points to track progress and trends in coverage for the outcome indicators. In addition, through multivariate analysis models, we accounted for possible demographic confounders on the outcome of all core indicators. Furthermore, the LQAS method used in this study was a robust rapid methodology in assessing coverages when distribution assumptions for a community during selection have been carefully carried out. The sampling method has potential to provide timely information to help re-design or adjust strategies.

## Conclusion

In conclusion, strengthening of community-based services in poor and remote districts through support to community health volunteers called SMAGs, were found to contribute to increasing coverage of maternal and neonatal health interventions. The observed findings in this study are suggestive of the important role SMAGs play as health promotion champions and community health systems agents for the poor and remote populations. This further suggests that strengthening community-based services among the poorest and remote populations can contribute to increasing access to and equitable coverage of MNH interventions notwithstanding the need for grounding in local knowledge systems and contexts. In addition, and considering that human resources for health continue to be a challenge in most developing countries, it seems reasonable to conclude that joint engagement of SMAGs with skilled healthcare providers could be a vital human resource complementary team approach which could, in turn, improve service delivery positively in sustainable and locally acceptable manner. Given this possibility, we, therefore, argue for context-specific and innovative task shifting strategies that exploit local knowledge systems and human resources like SMAGs with the potential to provide quality services among remote and marginalised settings where the need is greatest. Lastly but not the least, we recommend the need for further research to evaluate the effect of increased service utlisation rates on staff workload and quality of services in the intervention sites. Research is also needed to understand why coverage for some indicators in some districts had minimal improvement. In doing so we believe we could better understand the supply and demand interactions associated with improved MNCH services and in turn possibly be able to predict what could be happening strategically ranging from referral practices, socio-cultural barriers to system enablers in the provision of services among the SMAGs in rural and remote areas of Zambia.

## Supporting information

S1 File Household Survey Questionnaire—English version. (DOCX)

S2 File Household Survey Questionnaire–Local language (Bemba) version. (DOCX)
