# Correction: Evaluation of a community-based intervention to improve maternal and neonatal health service coverage in the most rural and remote districts of Zambia

**DOI:** 10.1371/journal.pone.0200324

**Published:** 2018-07-02

**Authors:** Choolwe Jacobs, Charles Michelo, Mumbi Chola, Nicholas Oliphant, Hikabasa Halwiindi, Sitali Maswenyeho, Kumar Sridutt Baboo, Mosa Moshabela

There is an error in the title of Table 3. The correct title is: Results of the logistic regression analysis of the factors related to the endline outcome of use of the three of the selected core indicators (ANC at least four times, SBA, PNC within 48 hours) over a three year time period.

There are errors in Tables 1 and 3. The *p* values in both tables are incorrect. Please see the correct Tables 1 and 3 here.

**Table 1:** Characteristics of mothers at Baseline, Midline and Endline surveys for the intervention.

Variable	Baseline (n = 551) %(95% CI)	Midline (n = 550) %(95% CI)	Endline (n = 551) %(95% CI)	***p value
**Individual Characteristics**				
**Age**				
15–24	40 (36–46)	41 (36–45)	43 (38–47)	0.517
25 and above	60 (56–64)	59 (55–63)	57 (52–61)	
**Marital Status**				
Not married	35 (32–38)	11 (9–14)	13 (10–16)	<0.001
Married	65 (62–68)	89 (86–91)	87 (84–90)	
**Ever attended school**				
No	20 (16–23)	22 (18–25)	17(13–19)	0.117
Yes	80 (77–84)	78 (74–81)	83 (81–87)	
**Level of education**				
Never been to school	21(17–24)	25 (21–29)	20 (17–24)	
Incomplete primary	53 (48–57)	42 (38–46)	42 (38–47)	
Complete primary	16 (13–19)	14(12–18)	15 (12–18)	
Post Primary*	10 (8–13)	19 (16–22)	21 (18–25)	<0.001
**Women’s Literacy**				
Cannot read at all	63 (58–68)	63 (58–67)	59 (55–63)	
Can read**	37 (33–41)	37 (33–41)	41 (37–45)	0.244
**Received Messages on birth preparedness**				
No	##	16 (13–19)	10 (7–12)	
Yes	##	84 (81–87)	90 (87–93)	<0.001
**Received Messages on birth preparedness from SMAG/CHW**				
No	##	49 (37–47)	31 (27–35)	
Yes	##	51 (53 - .62)	69 (64–73)	<0.001
**Community Characteristics**				
**Presence of a trained SMAG/CHW within the community**				
**No**	##	53 (48–58)	30 (25–36)	
Yes	##	47 (43–51)	70 (66–74)	<0.001
**Distance to the health facility**				
Within 5 Km	##	47 (43–52)	48 (43–52)	
Beyond 5 km	##	53 (48–57)	52 (48–56)	0.975

* Post primary means attempted and completed secondary;

^##^ Variable not measured at baseline

**Can read included those that could read part of the sentence;

***p value calculated using the Pearson’s chi square test

**Table 3:** Results of the logistic regression analysis of the factors related to the endline outcome of use of the three selected core indicators (ANC at least four times, SBA, PNC with 48 hours by a SMAG) over a three year time period.

Variables	Proportions N = 551% (n)**	ANC at least 4 times		Skilled Birth Attendance		postnatal care	
		Adjusted Odds Ratio (95% CI)	P-value	Adjusted Odds Ratio (95% CI)	P-value	Adjusted Odds Ratio (95% CI)	P-value
**Mother’s Literacy**							
Cannot read at all		1		1		1	
Can read*		1.40 (0.96–2.03)	0.081	1.44 (1.14–1.99)	0.028	1.59 (1.17–2.66)	0.030
**Distance to the health facility**							
Within 5 Km	48 (262)	1		1		1	
Beyond 5 km	52 (289)	0.58 (0.45–0.75)	<0.001	0.62 (0.47–0.81)	<0.001	1.03 (0 .79–1.35)	0.126
**Received HIV test Results during Pregnancy**							
No	15 (84)			1		1	
Yes	85 (465)	#	#	1.72 (1.13–2.61)	0.012	2.04 (0.95–4.37)	0.068
**Presence of a trained SMAG within the community**							
No		1		1		1	
Yes		1.20 (0.93–1.56)	0.160	1.29 (0.96–1.72)	0.096	1.86 (1.26–2.73)	0.002
**‘Received Messages on birth preparedness from SMAG**	31 (158)						
No	69 (349)	1		1		1	
Yes		1.70 (0.97–2.99)	0.065	1.49 (1.10–2.00)	0.009	1.76 (1.20–2.19)	0.004
**Received one ANC visit provided by skilled provider**							
No	33 (181)			1		1	
Yes	67 (366)	#		4.01 (2.88–5.72)	<0.001	2.11 (1.49–3.68)	0.008
**Skilled Birth Attendance**							
No	50 (276)					1	
Yes	50 (273)	#		#		2.41 (1.82–3.15)	<0.001

^#^ Predictor variable not included in the model for the individual outcome variable

*Women that could read the whole sentence or part of the sentence;

**Coverage at endline
