# Supplementary material for: Evaluation of a community-based intervention to improve maternal and neonatal health service coverage in the most rural and remote districts of Zambia
Source: PLoS One. 2018 Jan 16;13(1):e0190145. doi: 10.1371/journal.pone.0190145 (PMC5770027; doi:10.1371/journal.pone.0190145)
Supplement: S1 File — (DOCX) [file pone.0190145.s001.docx]

**HOUSEHOLD QUESTIONNAIRE FOR ELECTRONIC VERSION – ENGLISH VERSION**

**Survey Name: 0_5_months_Zambia**

**No of Questions: 69**

=========================================================================

**1:1. ZAMBIA HPP LQAS**

**MOTHERS OF CHILDREN 0-5 MONTHS (label)**

**2:2. [SELECT THE SURVEY] (multi)**

Data Field Name: SURVEY

Possible responses:

- BASELINE

- MIDLINE

- ENDLINE

**3:3. [INSTRUCTIONS FOR THE DATA COLLECTOR ARE IN [PARENTHESES]. DO NOT READ RESPONSE OPTIONS TO THE MOTHER UNLESS THE QUESTION TELLS YOU TO DO SO. REMEMBER TO USE YOUR INTERVIEW SKILLS.] (Label)**

**4:4. [SECTION 1: QUESTIONS FOR THE DATA COLLECTOR] (label)**

**5:5. [RECORD THE LQAS QUESTIONNAIRE NUMBER OUT OF 19] (numeric)**

Data Field Name: LQAS_NUMBER

**6:6. [RECORD THE SUPERVISION AREA NUMBER] (numeric)**

Data Field Name: SA

**7:7. [SELECT THE NAME OF THE PROVINCE] (multi)**

Data Field Name: PROVINCE

Possible responses:

- LUAPULA

- NORTHERN

**8:8. [SELECT THE NAME OF THE DISTRICT. SCROLL DOWN TO SEE ALL OPTIONS] (multi)**

Data Field Name: DISTRICT

Possible responses:

- CHIENGE

- LUWINGU

- MUNGWI

- SAMFYA

**9:9. [RECORD THE NAME OF THE COMMUNITY] (text)**

Data Field Name: COMMUNITY

**10:10. [RECORD THE NAME OF THE NEAREST HEALTH FACILITY] (text)**

Data Field Name: HEALTH_FACILITY

**11:11. [RECORD THE DISTANCE IN KILOMETERS FROM THIS COMMUNITY TO THE NEAREST HEALTH FACILITY] (multi)**

Data Field Name: KM_HEALTH_FACILITY

Possible responses:

- 0-5 KM

- MORE THAN 5 KM

**12:12. [RECORD WHETHER A SAFE MOTHERHOOD ACTION GROUP (SMAG) WORKS IN THIS COMMUNITY] (multi)**

Data Field Name: SMAG

Possible responses:

- YES

- NO

**13:13. [RECORD THE DATA COLLECTOR'S (YOUR) NAME] (text)**

Data Field Name : DATA_COLLECTOR

**14:14. [RECORD THE DATE OF INTERVIEW] (date)**

Data Field Name : DATE

**15:15. [SECTION 2: CONSENT] (label)**

**GREETINGS. MY NAME IS _________AND I WORK WITH ________DISTRICT. WE ARE CONDUCTING A SURVEY ABOUT MATERNAL AND NEWBORN HEALTH IN YOUR COMMUNITIES. YOU HAVE BEEN SELECTED RANDOMLY AND WE WOULD VERY MUCH APPRECIATE YOUR PARTICIPATION IN THIS SURVEY. THE INFORMATION YOU PROVIDE WILL HELP THE DISTRICT TO PLAN AND IMPROVE HEALTH SERVICES. THE INTERVIEW USUALLY TAKES ABOUT 30 MINUTES TO COMPLETE. WHATEVER INFORMATION YOU PROVIDE WILL BE KEPT CONFIDENTIAL AND WILL NOT BE SHOWN TO OTHER PERSONS.?**

**PARTICIPATION IN THIS SURVEY IS VOLUNTARY AND YOU CAN CHOOSE NOT TO ANSWER ANY INDIVIDUAL QUESTION OR ALL OF THE QUESTIONS. HOWEVER, WE HOPE THAT YOU WILL PARTICIPATE IN THIS SURVEY SINCE YOUR VIEWS ARE IMPORTANT. DO YOU AGREE TO PARTICIPATE IN THIS SURVEY? [IF NO, MARK THIS HOUSE AS A REFUSAL IN THE TABLE FOR SEQUENCE OF HOUSEHOLDS VISITED AND GO TO THE NEXT HOUSE] (multi)**

Data Field Name : CONSENT

Possible responses:

- YES

- NO

**16:16. WHAT IS THE FIRST LETTER OF YOUR FIRST NAME? AND WHAT IS THE FIRST LETTER OF YOUR SURNAME? [RECORD THE MOTHER'S INITIALS] (text)**

Data Field Name : MOTHER_INITIALS

**17:17. DO YOU HAVE A CHILD AGED 0-5 MONTHS OF AGE LIVING IN YOUR HOUSEHOLD? [VERIFY WITH THE CHILD VACCINATION CARD OR MATERNAL CARD IF POSSIBLE. RECORD WHETHER A CHILD 0-5 MONTHS LIVES IN THE HOUSEHOLD] (multi)**

Data Field Name : INFANT_0_5_MONTHS

Possible responses:

- YES

- NO

**18:18. HOW MANY INFANTS 0-5 MONTHS OF AGE LIVE IN THIS HOUSEHOLD? (numeric)**

Data Field Name: NUMBER_INFANTS

**19:19. [IF NO CHILD 0-5 MONTHS OF AGE, THANK THE MOTHER FOR HER TIME. GO TO NEXT NEAREST DOOR UNTIL YOU FIND A CHILD 0-5 MONTHS. START THE QUESTIONNAIRE AT CONSENT (QUESTION 17). IF MORE THAN ONE CHILD 0-5 MONTHS OF AGE, RANDOMLY SELECT 1 CHILD 0-5 MONTHS OF AGE] (label)**

**20:20. [RECORD THE NAME OF THE SELECTED CHILD] (text)**

Data Field Name: NAME_CHILD

**21:21. [SECTION 3: CHILD'S BACKGROUND] NOW I WOULD LIKE TO ASK YOU SOME QUESTIONS ABOUT [NAME'S] BACKGROUND (label)**

**22:22. WHAT IS THE SEX OF [NAME]? (multi)**

Data Field Name: SEX_CHILD

Possible responses:

- MALE

- FEMALE

**23:23. WAS [NAME] BORN IN 2013??**

**[CHECK THE CHILD HEALTH CARD OR MATERNAL ANTENATAL CARD IF POSSIBLE] (multi)**

Data Field Name: YEAR_CHILD_BORN

Possible responses:

- YES

- NO

**24:24. IN WHAT MONTH WAS [NAME] BORN? [SCROLL DOWN TO SEE ALL OPTIONS. CHECK THE CHILD HEALTH CARD OR ANTENATAL CARD IF POSSIBLE. IF CHILD WAS BORN BEFORE MARCH 2013, THE CHILD IS TOO OLD] (multi)**

Data Field Name : MONTH_CHILD_BORN

Possible responses:

- MARCH

- APRIL

- MAY

- JUNE

- JULY

- AUGUST

- SEPTEMBER

- OCTOBER

**25:25. [SECTION 4: MOTHER'S BACKGROUND] NOW I WOULD LIKE TO ASK YOU SOME QUESTIONS ABOUT YOUR BACKGROUND (label)**

**26:26. IN WHAT YEAR WERE YOU BORN? [CHECK THE NATIONAL REGISTRATION CARD IF POSSIBLE] (numeric)**

Data Field Name: MOTHER_YEAR_BORN

**27:27. HOW OLD ARE YOU IN COMPLETED YEARS? (numeric)**

Data Field Name: MOTHER_AGE_YRS

**28:28. HAVE YOU EVER ATTENDED SCHOOL? [IF NO, IT WILL SKIP AUTOMATICALLY] (multi)**

Data Field Name: MOTHER_SCHOOL

Possible responses:

- YES

- NO

**29:29. WHAT IS THE HIGHEST LEVEL OF SCHOOL YOU ATTENDED? [SCROLL DOWN TO SEE ALL OPTIONS] (multi)**

Data Field Name: MOTHER_HIGHEST_EDCUATION

Possible responses:

- PRESCHOOL (LESS THAN 1ST GRADE)

- INCOMPLETE PRIMARY (1ST-6TH GRADE)

- COMPLETE PRIMARY (7TH GRADE)

- INCOMPLETE SECONDARY (8TH-11TH GRADE)

- COMPLETE SECONDARY (12 GRADE)

- HIGHER

**30:30. NOW, I WOULD LIKE YOU TO READ THIS SENTENCE TO ME: ?**

**[SHOW SENTENCE ON PAPER TO RESPONDENT. SCROLL DOWN TO SEE THE WHOLE QUESTION]?**

**"I AM GOING HOME TO COOK FOOD"?**

**[IF RESPONDENT CANNOT READ WHOLE SENTENCE, PROBE]?**

**CAN YOU READ PART OF THE SENTENCE TO ME? (multi)**

Data Field Name: MOTHER_LITERACY

Possible responses:

- CANNOT READ AT ALL

- ONLY PARTS

- WHOLE SENTENCE

**31:31. WHAT IS YOUR CURRENT MARITAL STATUS? [SCROLL DOWN TO SEE ALL OPTIONS. READ RESPONSE OPTIONS AND RECORD THE RESPONSE GIVEN.] (multi)**

Data Field Name: MOTHER_MARITAL_STATUS

Possible responses:

- SINGLE, NO PARTNER

- SINGLE, NON REGULAR PARTNER

- SINGLE, REGULAR PARTNER

- MARRIED

- COHABITING

- WIDOWED

- DIVORCED/SEPARATED

**32:32. [SECTION 6: ANTENATAL CARE] NOW I WOULD LIKE TO ASK YOU QUESTIONS ABOUT ANTENATAL CARE DURING YOUR PREGNANCY WITH [NAME] (label)**

**33:33. DID YOU SEE ANYONE FOR ANTENATAL CARE DURING YOUR PREGNANCY WITH [NAME]? (multi)**

Data Field Name: ANY_ANC

Possible responses:

- YES

- NO

**34:34. FOR ANC DURING THIS PREGNANCY, WHOM DID YOU SEE? [PROBE] ANYONE ELSE? [SCROLL DOWN TO SEE ALL OPTIONS. DO NOT READ THE OPTIONS. PROBE FOR THE TYPE OF PERSON SEEN AND RECORD ALL ANSWERS GIVEN.]?**

**(multi)**

Data Field Name: ANC_PROVIDER

Possible responses:

- DOCTOR

- NURSE

- CLINICAL OFFICER

- MIDWIFE

- EHT

- COMMUNITY HEALTH WORKER

- TRADITIONAL BIRTH ATTENDANT

- OTHER HEALTH WORKER

**35:35. HOW MANY MONTHS PREGNANT WERE YOU WHEN YOU FIRST RECEIVED ANTENATAL CARE FOR THIS PREGNANCY? [SCROLL DOWN TO SEE ALL OPTIONS]?**

**(multi)**

Data Field Name: ANC1_TIMING

Possible responses:

- 2 MONTHS

- 3 MONTHS

- 4 MONTHS

- 5 MONTHS

- 6 MONTHS

- 7 MONTHS

- 8 MONTHS

- 9 MONTHS

- DON'T KNOW

**36:36. HOW MANY TIMES DID YOU RECEIVE ANTENATAL CARE DURING THIS PREGNANCY? [SCROLL DOWN TO SEE ALL OPTIONS] (multi)**

Data Field Name: ANC_TIMES

Possible responses:

- 1 TIME

- 2 TIMES

- 3 TIMES

- 4 TIMES

- MORE THAN 4 TIMES

- DON'T KNOW

**37:37. AS PART OF YOUR ANTENATAL CARE DURING THIS PREGNANCY, WERE ANY OF THE FOLLOWING DONE AT LEAST ONCE [SCROLL DOWN TO SEE ALL OPTIONS. READ THE OPTIONS AND RECORD ALL THE ACTIONS DONE AT ANC.] (multi)**

Data Field Name: ANC_CONTENT

Possible responses:

- YOUR BLOOD PRESSURE WAS MEASURED

- YOU GAVE A URINE SAMPLE

- YOU GAVE A BLOOD SAMPLE

- YOU WERE WEIGHED

- YOU WERE EXAMINED / ASSESSED

- NONE

**38:38. [SECTION 7: ANTIMALARIALS DURING ANTENATAL CARE] NOW I WOULD LIKE TO ASK YOU QUESTIONS ABOUT MEDICINE TO PREVENT MALARIA DURING PREGNANCY (label)**

**39:39. DURING ANY OF THESE ANTENATAL VISITS WHILE YOU WERE PREGNANT WITH [NAME], DID YOU TAKE ANY MEDICINE IN ORDER TO PREVENT YOU FROM GETTING MALARIA? [IF NO OR DON'T KNOW, IT WILL SKIP AUTOMATICALLY]. (multi)**

Data Field Name: ANTIMALARIAL_PREGNANCY

Possible responses:

- YES

- NO

- DON'T KNOW

**40:40. WHICH MEDICINES DID YOU TAKE TO PREVENT MALARIA? [SCROLL DOWN TO SEE ALL OPTIONS. DO NOT READ THE OPTIONS. RECORD ALL MEDICINES TAKEN. IF TYPE OF MEDICINE IS NOT DETERMINED, SHOW TYPICAL ANTI-MALARIAL TO RESPONDENT. IF SP (FANSIDAR) WAS NOT TAKEN, IT WILL SKIP AUTOMATICALLY]. (multi)**

Data Field Name: ANTIMALARIAL_TYPE_PREGNANCY

Possible responses:

- SP (FANSIDAR)

- COARTEM

- QUININE

- OTHER

- DON'T KNOW

**41:41. DURING THIS PREGNANCY, HOW MANY TIMES DID YOU TAKE SP (FANSIDAR)? [IF THEY DON'T KNOW, RECORD 88]. (numeric)**

Data Field Name: FANSIDAR_TIMES

**42:42. HOW MANY TABLETS OF SP (FANSIDAR) WERE YOU GIVEN DURING YOUR PREGNANCY WITH [NAME]? [IF THEY DON'T KNOW, RECORD 88]. (numeric)**

Data Field Name: FANSIDAR_TABLETS_NUMBER

**43:43. [SECTION 9: BIRTH PREPAREDNESS] NOW I WOULD LIKE TO ASK YOU QUESTIONS ABOUT BIRTH PREPAREDNESS (label)**

**45:45. HAVE YOU HEARD MESSAGES ABOUT BIRTH PREPAREDNESS IN THE PAST SIX MONTHS? [IF NO OR DON'T NO, IT WILL SKIP AUTOMATICALLY] (multi)**

Data Field Name: BIRTHPREPAREDNESS_MESSAGES

Possible responses:

- YES

- NO

- DON'T KNOW

**46:46. WHAT/WHO PROVIDED THESE MESSAGES? [SCROLL DOWN TO SEE ALL OPTIONS] (multi)**

Data Field Name: BIRTHPREPAREDNESS_MESSAGES_PROVIDER

Possible responses:

- SMAG (SAFE MOTHERHOOD ACTION GROUP)

- COMMUNITY HEALTH WORKER

- DOCTOR / NURSE / MIDWIFE

- RADIO

- TELEVISION

- OTHER

**47:47. [SECTION 11: SKILLED BIRTH ATTENDANCE, FACILITY DELIVERY AND POSTNATAL CARE] NOW I WOULD LIKE TO ASK YOU SOME QUESTIONS ABOUT THE DELIVERY OF [NAME], WHERE YOU GAVE BIRTH TO [NAME], AND CHECKS ON [NAME'S] HEALTH AND YOUR HEALTH AFTER YOU GAVE BIRTH (label)**

**48:48. WHO ASSISTED WITH THE DELIVERY OF [NAME]? [SCROLL DOWN TO SEE ALL OPTIONS. DO NOT READ OPTIONS. Probe] ANYONE ELSE? [PROBE FOR THE TYPE OF PERSON ASSISTING AND RECORD ALL ANSWERS GIVEN. IF RESPONDENT SAYS NO ONE ASSISTED, PROBE TO DETERMINE WHETHER ANY ADULTS WERE PRESENT AT THE DELIVERY.] (multi)**

Data Field Name: ASSISTED_DELIVERY_PERSON

Possible responses:

- DOCTOR

- NURSE

- CLINICAL OFFICER

- MIDWIFE

- EHT

- TRADITIONAL BIRTH ATTENDANT

- COMMUNITY HEALTH WORKER

- OTHER HEALTH WORKER

- RELATIVE / FRIEND

- OTHER ADULT

- OTHER

- NO ONE

- DON'T KNOW

**49:49. WHERE DID YOU GIVE BIRTH FROM [NAME]? [SCROLL DOWN TO SEE ALL OPTIONS. PROBE TO IDENTIFY THE TYPE OF SOURCE] (multi)**

Data Field Name: PLACE_DELIVERY

Possible responses:

- RESPONDENT'S HOME

- OTHER HOME

- GOVERNMENT HOSPITAL

- GOVERNMENT CLINIC/HEALTH CENTER

- GOVERNMENT HEALTH POST

- MISSION HOSPITAL / CLINIC

- OTHER PUBLIC

- PRIVATE HOSPITAL

- PRIVATE CLINIC

- PRIVATE MATERNITY HOME

- OTHER PRIVATE

- OTHER

**50:50. [THIS IS AN AUTOMATIC VERIFICATION OF FACILITY DELIVERY. THE RESPONSE IS AUTOMATICALLY CALCULATED. YOU DO NOT HAVE TO DO ANYTHING. SCROLL TO THE NEXT QUESTION] (multi)**

Data Field Name: VERIFICATION_FACILITY_DELIVERY

Possible responses:

- FACILITY DELIVERY

- NOT A FACILITY DELIVERY

**51:51. [RECORD YES FOR THIS QUESTION. YOU DO NOT HAVE TO ASK THE MOTHER ANYTHING. JUST RECORD YES AND SCROLL TO THE NEXT QUESTION. IT WILL SKIP AUTOMATICALLY IF NECESSARY] (multi)**

Data Field Name: FACILITY_SKIP1

Possible responses:

- YES

- NO

**52:52. NOW I WOULD LIKE TO ASK YOU SOME QUESTIONS ABOUT WHAT HAPPENED IN THE HOURS AND DAYS AFTER BIRTH OF [NAME].**

**YOU HAVE SAID THAT YOU GAVE BIRTH IN [NAME OR TYPE OF FACILITY]. HOW LONG DID YOU STAY THEREAFTER THE DELIVERY? [SCROLL DOWN TO SEE ALL OPTIONS] (multi)**

Data Field Name: LENGTH_FACILITY_STAY

Possible responses:

- LESS THAN 6 HOURS

- 6-11 HOURS

- 12-23 HOURS

- 1-2 DAYS

- 3 DAYS OR MORE

- DON'T KNOW

**53:53. NOW I WOULD LIKE TO TALK TO YOU ABOUT WHAT HAPPENED AFTER YOU LEFT THE FACILITY YOU DELIVERED IN. DID ANYONE CHECK ON [NAME'S] HEALTH AFTER YOU LEFT THE TYPE OF FACILITY THEY DELIVERED IN? [THERE IS AN AUTOMATIC SKIP FOR YES OR FOR NO] (multi)**

Data Field Name: NEWBORN_PNC_POSTDISCHARGE

Possible responses:

- YES

- NO

**54:54. I WOULD LIKE TO TALK TO YOU ABOUT CHECKS ON [NAME]’S HEALTH AFTER DELIVERY – FOR EXAMPLE, SOMEONE EXAMINING [NAME], CHECKING THE CORD, OR SEEING IF THE BABY IS OK. AFTER [NAME] WAS DELIVERED, DID ANYONE CHECK ON HIS/HER HEALTH? [IF NO, IT WILL SKIP AUTOMATICALLY] (multi)**

Data Field Name: NEWBORN_PNC_NONFACILITY_NONASSISTED

Possible responses:

- YES

- NO

**54:54. DID SUCH A CHECK HAPPEN ONLY ONCE, OR MORE THAN ONCE? [IF MORE THAN ONCE, IT WILL AUTOMATICALLY SKIP] (multi)**

Data Field Name: NEWBORN_PNC_NONFACILITY_NONASSISTED_TIMES

Possible responses:

- ONCE

- MORE THAN ONCE

**55:55. HOW LONG AFTER DELIVERY DID THAT CHECK HAPPEN? [SCROLL DOWN TO SEE ALL OPTIONS] (multi)**

Data Field Name: NEWBORN_TIMING_FIRST_PNC_ONLY_1

Possible responses:

- LESS THAN 1 HOUR AFTER BIRTH

- 1-23 HOURS AFTER BIRTH

- 1-2 DAYS AFTER BIRTH

- 3-6 DAYS AFTER BIRTH

- 7 DAYS OR MORE AFTER BIRTH

**56:56. HOW LONG AFTER DELIVERY DID THE FIRST OF THESE CHECK HAPPEN? [SCROLL DOWN TO SEE ALL OPTIONS] (multi)**

Data Field Name: NEWBORN_TIMING_FIRST_PNC_MORETHAN_1

Possible responses:

- LESS THAN 1 HOUR AFTER BIRTH

- 1-23 HOURS AFTER BIRTH

- 1-2 DAYS AFTER BIRTH

- 3-6 DAYS AFTER BIRTH

- 7 OR MORE DAYS AFTER BIRTH

**57:57. WHO CHECKED ON [NAME'S] HEALTH AT THAT TIME? [SCROLL DOWN TO SEE ALL OPTIONS] (multi)**

Data Field Name: NEWBORN_PNC1_PERSON

Possible responses:

- DOCTOR

- NURSE

- CLINICAL OFFICER

- MIDWIFE

- EHT

- SMAG

-TRADITIONAL BIRTH ATTENDANT

- COMMUNITY HEALTH WORKER

- OTHER HEALTH WORKER

- RELATIVE / FRIEND

- OTHER

**58:58. DURING THIS CHECK, WAS THE FOLLOWING DONE FOR [NAME]? [SCROLL DOWN TO SEE ALL OPTIONS. READ THE OPTIONS AND RECORD THE MOTHER'S RESPONSES]. (multi)**

Data Field Name: NEWBORN_SIGNAL_FUNCTIONS_NONFACILITY_

Possible responses:

- EXAMINE CORD

- COUNSEL YOU ON DANGER SIGNS FOR NEWBORNS

- ASSESS THE TEMPERATURE OF YOUR BABY

- COUNSEL YOU ON BREASTFEEDING AND OBSERVE YOUR BABY BREASTFEEDING

- WEIGH THE BABY

- IDENTIFICATION AND REFERRAL OF ILLNESS

- EDUCATION ON HOME CARE FOR THE SICK INFANT

- EDUCATION ON HYGIENE, HAND WASHING AND SANITATION

**59:59. AFTER THE DELIVERY WAS OVER AND THE PERSON THAT ASSISTED THE DELIVERY LEFT, DID ANYONE CHECK ON YOUR HEALTH? [THERE IS AN AUTOMATIC SKIP] (multi)**

Data Field Name: MATERNAL_PNC_POST_SBA

Possible responses:

- YES

- NO

**60:60. AFTER THE BIRTH OF [NAME], DID ANYONE CHECK ON YOUR HEALTH? I MEAN SOMEONE ASSESSING YOUR HEALTH, FOR EXAMPLE ASKING QUESTIONS ABOUT YOUR HEALTH OR EXAMINING YOU. [THERE IS AN AUTOMATIC SKIP]?**

**(multi)**

Data Field Name: MATERNAL_PNC_NO_SBA

Possible responses:

- YES

- NO

**61:61. DID SUCH A CHECK HAPPEN ONLY ONCE, OR MORE THAN ONCE? (multi)**

Data Field Name: MATERNAL_PNC_POST_SBA_TIMES

Possible responses:

- ONCE

- MORE THAN ONCE

**62:62. HOW LONG AFTER DELIVERY DID THAT CHECK HAPPEN? (multi)**

Data Field Name: MATERNAL_TIMING_FIRST_PNC_ONLY_1

Possible responses:

- LESS THAN 1 HOUR AFTER BIRTH

- 1-23 HOURS AFTER BIRTH

- 1-2 DAYS AFTER BIRTH

- 3-6 DAYS AFTER BIRTH

- 7 DAYS OR MORE AFTER BIRTH

**63:63. HOW LONG AFTER DELIVERY DID THE FIRST OF THESE CHECK HAPPEN? (multi)**

Data Field Name : MATERNAL_TIMING_FIRST_PNC_MORETHAN_1

Possible responses:

- LESS THAN 1 HOUR AFTER BIRTH

- 1-23 HOURS AFTER BIRTH

- 1-2 DAYS AFTER BIRTH

- 3-6 DAYS AFTER BIRTH

- 7 DAYS OR MORE AFTER BIRTH

**64:64. WHO CHECKED ON YOUR HEALTH AT THAT TIME? (multi)**

Data Field Name: MATERNAL_PNC1_PERSON

Possible responses:

- DOCTOR

- NURSE / CLINICAL OFFICER

- MIDWIFE

- EHT

- SMAG

-TRADITIONAL BIRTH ATTENDANT

- COMMUNITY HEALTH WORKER

- OTHER HEALTH WORKER

- RELATIVE / FRIEND

- OTHER ADULT

**65:65. [SECTION 13: HIV TESTING] NOW I WOULD LIKE TO ASK YOU SOME QUESTIONS ABOUT HIV TESTING AND COUNSELING (label)**

**66:66. WERE YOU OFFERED AN HIV TEST DURING YOUR PREGNANCY WITH [NAME] AS PART OF YOUR ANTENATAL CARE WITH [NAME]? [IF NO THERE IS AN AUTOMATIC SKIP] (multi)**

Data Field Name: OFFERED_HIV_TEST

Possible responses:

- YES

- NO

**67:67. I DON'T WANT TO KNOW THE RESULTS, BUT WERE YOU TESTED FOR HIV AS PART OF YOUR ANTENATAL CARE WITH [NAME]? (multi)**

Data Field Name: TOOK_HIV_TEST

Possible responses:

- YES

- NO

- DON'T KNOW

**68:68. I DON'T WANT TO KNOW THE RESULTS, BUT DID YOU RECEIVE THE RESULT OF YOUR HIV TEST AS PART OF YOUR ANTENATAL CARE? [IF NO THERE IS AN AUTOMATIC SKIP] (multi)**

Data Field Name: RECEIVED_RESULTS_HIV_TEST

Possible responses:

- YES

- NO

- DON'T KNOW

**69:69. I DON'T WANT TO KNOW THE RESULTS, BUT WERE YOU COUNSELED ON THE RESULTS OF YOUR HIV TEST AS PART OF YOUR ANTENATAL CARE? (multi)**

Data Field Name: RECEIVED_RESULTS_COUNSELED_HIV_TEST

Possible responses:

- YES

- NO

- DON'T KNOW

**[THANK THE MOTHER FOR HER TIME AND PARTICIPATION] (label)**
