# Supplementary material for: Evaluation of a community-based intervention to improve maternal and neonatal health service coverage in the most rural and remote districts of Zambia
Source: PLoS One. 2018 Jan 16;13(1):e0190145. doi: 10.1371/journal.pone.0190145 (PMC5770027; doi:10.1371/journal.pone.0190145)
Supplement: S2 File — (DOCX) [file pone.0190145.s002.docx]

**HOUSEHOLD QUESTIONNAIRE FOR ELECTRONIC VERSION – BEMBA VERSION**

**Survey Name: 0_5_months_Zambia**

**No of Questions: 69**

=========================================================================

**1:1. ZAMBIA HPP LQAS**

**BANYINEFWE ABALI NABANA UKUFUMA PA MYENSHI 0-5 (****Saleni)**

**2.2. [SALENI UKUFWELISHA] Ubwingi**

Data Field Name: Ukufwelisha

**Ukufwelisha:**

- KWA KUBALILAPO
- KWA KOKELEPO NANGULA PA KATI
- KWA KU LEKELESHA

**3:3. [IFILANGILILO FYA BANTU BALESENDA ILYASHI FILI MU [PARENTHESES]. MWIBELENGA IFILANGILILO FYA AMASUKO KULI NACIFYASHI KANOFYE ILIPUSHO LYAMYEBA UKUTI MUBELENGE. IBUKISHENI UKUBOFYA AMANO MWASAMBILILA PALWA KWIPUSHA.] (Saleni)**

**4:4. [ICIPANDWA 1: AMEPUSHO YA MUNTU ULESENDA ILYASHI] (Saleni)**

**5:5. [LEMBENI INAMBALA YA BWIPUSHO YA LQAS AMEIPUSHO UKUFUMA PAYALI 19] (ifipendwa)**

Data Field Name: LQAS_INAMBA

**6:6. [LEMBENI INAMBA YA CITUNGU ICILETUNGULILA] (ifipendwa)**

Data Field Name: SA

**7:7. [LEMBENI ISHINA YA CITUNGU] (ubwingi)**

Data Field Name: ICITUNGU

Amasuko Mwingasalapo:

- KU LUAPULA

- KU NORTHERN

**8:8. [SALENI ISHINA YA ICITUNGU. PITENEMO PAKUTI MUMONEMO AMASUKO YONSE AYALIPO] (ubwingi)**

Data Field Name: ICITUNGU

Amasuko Mwingasalapo:

- KU CHIENGE

- KU LUWINGU

- KU MUNGWI

- KU SAMFYA

**9:9. [LEMBENI ISHINA YA NCHENDE] (ifilebo)**

Data Field Name: INCHENDE

**10:10. [LEMBENI ISHINA YA CHIPATALA ICILI MUPEPI] (ifilembo)**

Data Field Name: ICHIPATALA

**11:11. [LEMBENI INTAMFU MUMA KILOMETERS UKUFUMA PANCHENDE UKUFIKA PACIPATALA ICILI MUPEPI] (ubwingi)**

Data Field Name: KM_HEALTH_FACILITY

Amasuko Mwingasalapo:

- UKUFUMA 0-5 KM

- UKUPITILLILA 5 KM

**12:12. [LEMBENI NGACAKUTILA MUNCHENDE MWALIBA AKABWUNGWE KA SAFE MOTHERHOOD ACTION GROUP (SMAG) (ubwingi)**

Data Field Name: SMAG

Amasuko Mwingasalapo:

- EMUKWAI

- IYO

**13:13. [LEMBENI INSHINA YA ULESENDA IFISHINKA (ISHINA YENU)] (ifilembo)**

Data Field Name : DATA_COLLECTOR

**14:14. [LEMBENI UBUSHIKU MUSENDA IFISHINKA] (ubushiku)**

Data Field Name : UBUSHIKU

**15:15. [ICIPANDWA 2: UKUSUMINISHA] (Saleni)**

**NAMIPOSHA. ISHINA YA NDI NINE _________ELYO MBOMBA NABA ________MUCHITUNGU. TULESENDA IFISHINKA PALWA BANACIFYASHI NABANA MU CHITUNGU CHENU. NAMUSALWA UKUSENDAMO ULUBALI ELYO NATUTEMWA UKUTI MWASENDAMO ULUBALI MUKUFWALISHA UKU. ILISHI MWALATUPELA IKAFWA ICITUNGU CENU UKUBIKAKO ISHILA ISHISUMA ISHABUMI. UKU KULANSHINA KWASENDA INSHITA YA BAMINETI 30. ILYASHI LYONSE MWALATWEBA TWALASUNGA MUBUMFUSOLO ELYO TATWALANGE UMUNTU NANGU UMO?**

**UKUSENDAMO ULUBALI MUKUFWILISHA UKU KWAKUIFWALAFYE KWENU ELYO KUTI MWASALA UKUKANA YASUKA AMEPUSHO AYALI YONSE. KOMA, TULI NECHICHETEKELO UKUTI MWALASENDEMO ULUBALI MUKUFWILISHA UKU UMULANDU WAKUTI IFYO MWALATWEBA FYACHIKANKALA.**

**BUSHE MUSEMISHA UKUSENDAMO ULUBALI? [NGA CHAKUTI BAKANA, LEMBENI IYI NG'ANDA NGA BAKANE MUCHIPANDWA CHA MAYANDA AYO MUPITILEMO ELYO MUKONKANYEPO UKUYA PANG'ANDA IMBI] (ubwingi)**

Data Field Name : UKUSUMISHA

Amasuko Mwingasalapo:

- EMUKWAI

- IYO

**16:16. BUSHE NIKALANTA NSHI WANKUNTULO WESHINA YENU? ELYO NIKALANTA NSHI WAKUBALILAPO KWISHINA YABAFYASHI BENU? [LEMBENI IFYANKUNTULO KWESHINA LYA KWANACHIFYASHI] (ifilembo)**

Data Field Name : MOTHER_INITIALS

**17:17. BUSHE MWALIKWATA UMWANA YAMYENSHI UKUFUMA PA 0-5 ULI MUNG'ANDA ALEIKALA NENU? [FWAILISHENI NGACHAKUTI UMWANA ALICHINGILILWA KU MALWELE UKUBOMFYA VACCINATION CARD ATEMWA MATERNAL CARD. LEMBENI NGACHAKUTI MULI UMWANA YA MYENSHI PAKATI KA 0-5 UWIKALA MUNG'ANDU UMU] (ubwingi)**

Data Field Name : INFANT_0_5_MONTHS

Amasuko Mwingasalapo:

- EMUKWAI

- IYO

**18:18. BUSHE NIBANGA ABANA UKUFUMA PA MYENSHI 0-5 ABEKALA MULI NG'ANDA? (ifipendwa)**

Data Field Name: NUMBER_INFANTS

**19:19 [NGACHAKUTI TAMULI UMWANA UWA MYENSHI 0-5, TOTELENI NACHIFYASHI PANSHITA YABO. KABIYENI PANG'ANDA IKONKELOPO PANKA MUSANGE UMWANA WA MYENSHI UKUFUMA 0-5. YAMBILENI AMEPUSHO UKUFUMA PAKU SUMINISHA (ILIPUSHO 17). NGA PALI ABANA ABENGI ABALI NEMYENSHI YAKUFYALWA 0-5, SALENIPOFYE UMWANA UMO ULI NEMYENSHI YAKUFYALWA 0-5] (Saleni)**

**20:20. [LEMBENI ISHINA YAMWANA MWASALA] (ifilembo)**

Data Field Name: NAME_CHILD

**21:21. [ICIPANDWA 3: IFISHINKA PA MWANA] NOMBA NDEFWAYA UKUMIPUSHA PAFISHINKA [AMASHINA] (Saleni)**

**22:22. BUSHE MWANANSHI [INSHINA]? (ubwingi)**

Data Field Name: SEX_CHILD

Amasuko Mwingasalapo:

- MALE

- FEMALE

**23:23. BUSHE [ISHINA] AFYELWE MU 2013?**

**[CHECHETENI MUCHIPEPELA CHAKUCHIPATALA CAMWANA NANGULA MATERNAL ANTENATAL CARD] (ubwingi)**

Data Field Name: YEAR_CHILD_BORN

Amasuko Mwingasalapo:

- EMUKWAI

- IYO

**24:24. BUSHE MWENSHI NSHI [ISHINA] AFYALILWEMO? [SALENI PAMASUKO YONSE ALIPO. CHECHETENI MUCHIPEPELA CHAKUCHIPATALA CAMWANA NANGULA MATERNAL ANTENATAL CARD. NGACHAKUTILA AFYELWE PANUMA YAMWENSHI WA MARCH 2013, UMWANA NISHI MUKALAMBA] (ubwingi)**

Data Field Name : MONTH_CHILD_BORN

Amasuko Mwingasalapo:

- MU MARCH

- MU APRIL

- MU MAY

- MU JUNE

- MU JULY

- MU AUGUST

- MU SEPTEMBER

- MU OCTOBER

**25:25. [ICHIANDWA 4: IFISHIKA PALI NACHIFYASHI] NOMBA NDEFYA UKWIPUSHA PALI NACHIFYASHI (Saleni)**

**26:26. MWAFYELWE MU MUMWAKA NSHI? [CHECHETENI PALI CHITUPA NGA EPOILI] (ifipendwa)**

Data Field Name: MOTHER_YEAR_BORN

**27:27. MULI NEMWAKA INGA? (ifipendwa)**

Data Field Name: MOTHER_AGE_YRS

**28:28. BUSHE MWALIYAPO KUSUKULU? [NGA IYO, YALATOLOKA IINE] (ubwingi)**

Data Field Name: MOTHER_SCHOOL

Amasuko Mwingasalapo:

- EMUKWAI

- IYO

**29:29. BUSHE MAPELELA MWISA MUMASAMBILILO YENU? [SALENIPO PAMASUKO YONSE AYALIPO] (ubwingi)**

Data Field Name: MOTHER_HIGHEST_EDCUATION

Amasuko Mwingasalapo:

- KU PRESCHOOL (LESS THAN 1ST GRADE)

- INCOMPLETE PRIMARY (1ST-6TH GRADE)

- COMPLETE PRIMARY (7TH GRADE)

- INCOMPLETE SECONDARY (8TH-11TH GRADE)

- COMPLETE SECONDARY (12 GRADE)

- HIGHER

**30:30. NOMBA NDEFWAYA MUMBELENGELE IFI: ?**

**[LANGENI IIPEPELA CHAFILEMBO KUBALESENDAMO ULUBALI. SALENI PAMEPUSHO YONSE AYALIPO]?**

**"NDEYA KUNG'ANDA MUKWIPIKA IFYAKULYA"?**

**[NGACHAKUTI ABALESENDAMO ULUBALI TABAKWANISHE UKUBELENGE FYONSE, FWAYENI INSHILA IMBI]?**

**BUSHE KUTI MWABELENGAPO FIMO? (ubwingi)**

Data Field Name: MOTHER_LITERACY

Amasuko Mwingasalapo:

- TETI BABELENGA NANGULA PANONO

- MUTUFIPYA FYE

- IFILEMBO FYONSE

**31:31. BUSHE MULYUPWA? [SALENI PAMASUKO YONSE AYALIPO. BELENGENI AMASUKO AYALIPO ELYO LEMBENI AMASUKO BALEMIPELA.] (ubwingi)**

Data Field Name: MOTHER_MARITAL_STATUS

Amasuko Mwingasalapo:

- NABA NEKA, NSHAKWATA UMUTEMWIKWA

- NABA NEKA, TAKWABA UMUTEMWIKWA

- NABA NEKA, NOTEMWIKA

- NALYUPWA

- NJIKALAFYE PAMO

- NALIFWILWA

- IFUPO FYALIPWA/TWALILEKANA

**32:32. [ICHIANDWA 6: ANTENATAL CARE] NOMBA NDEFWAYA UKWIPUSHA PALI ANTENATAL CARE ILYO MWALI NEFUMO LYA KWA [ISHINA] (Saleni)**

**33:33. BUSHE MWALIMONANAPO UMUNTU ULIONSE PALWA ANTENATAL CARE ILYO MWALI NEFUMO LYAKWA [ISHINA]? (ubwingi)**

Data Field Name: ANY_ANC

Amasuko Mwingasalapo:

- EMUKWAI

- IYO

**34:34. PALWA ANC ELYO MWALI NEFUMO, NANI MWAMONENE? [FWAILISHENI] NANI UMBI? [SALENI PAMASUKO AYALIPO. MWIBELENGA AMASUKO AYALIPO. FWAILISHENI PAMUNTU BAMWENE ELYO LEMBENI AMASUKO BAPELA.]?**

**(ubwingi)**

Data Field Name: ANC_PROVIDER

Amasuko Mwingasalapo:

- BA DOCTOR

- BA NURSE

- BA CLINICAL OFFICER

- BA MIDWIFE

- BA EHT

- BA COMMUNITY HEALTH WORKER

- BA TRADITIONAL BIRTH ATTENDANT

- BAMBI BA HEALTH WORKER

**35:35. BUSHE MWALI NEMYENSHI INGA ELYO MWAILE MUKUPIMISHA IFUMO PAMUKU WAKUBALILAPO? [SALENI PAMA SUKO YONSE AYALIPO]?**

**(ubwingi)**

Data Field Name: ANC1_TIMING

Amasuko Mwingasalapo:

- 2 MONTHS

- 3 MONTHS

- 4 MONTHS

- 5 MONTHS

- 6 MONTHS

- 7 MONTHS

- 8 MONTHS

- 9 MONTHS

- NSHISHIBE

**36:36. BUSHE MIKUINGA MWAPOKELELE ANTENATAL CARE PANSHITA YEFUMO IYI? [SALENI PAMASUKO YONSE AYALIPO] (ubwingi)**

Data Field Name: ANC_TIMES

Amasuko Mwingasalapo:

- 1 TIME

- 2 TIMES

- 3 TIMES

- 4 TIMES

- UKUPITILILA 4 TIMES

- NSHISHIBE

**37:37. NGA CHIMO PALWA ANTENATAL CARE YENU PANSHITA FUMO IYI, BUSHE PALI IFI FYALICHITEKEKO PAKASHITA KAMO [SALENI PAMASUKO AYALIPO. BELENGENI AMASUKO AYALIPO ELYO MULEMBE AMASUKO PA PALWA ANC.] (ubwingi)**

Data Field Name: ANC_CONTENT

Amasuko Mwingasalapo:

- MWALIPIMWA BLOOD PRESSURE

- MWA LIPELA IMISU MUKUPIMWA

- MWALIPELA UMULOPA MUKUPIMWA

- MWALIPIMWA UKUFINA

- MWALICHECHETWA

- TAPALI

**38:38. [ICHIANDWA 7: ANTIMALARIALS PANSHITA YA ANTENATAL CARE] NOMBA NDEFWAYA UKWIPUSHA AMEPUSHO PA MITI UKUCINGILILA MALARIA ILYO MULI PABUKULU (Saleni)**

**39:39. PANSHITA YAKUYA KU ANTENATAL ELYO MWALI NEFUMO YA [ISHINA], BUSHE MWALINWUNINEPO UMUTI UKUCHINGILILA MALARIA? [NGA IYO NANGULA NSHISHIBE, YALATOLOKA YEKA]. (Saleni)**

Data Field Name: ANTIMALARIAL_PREGNANCY

Amasuko Mwingasalapo:

- EMUKWAI

- IYO

- NSHISHIBE

**40:40. MUTI NSHI MWANWINE UKUCHINGILILA MALARIA? [SALKENI PA MASUKO AYALIPO. MWIBELENGA AMASUKO AYALIPO. LEMBENI UMUTI ONSE MWANWINE. NGA UMUTI TAWISHIBIKE, LANGENI IMBI ANTI-MALARIAL KULI NACHIFYASHI NGANI SP (FANSIDAR) MWANWINE, YALATOLOKA IINE]. (Saleni)**

Data Field Name: ANTIMALARIAL_TYPE_PREGNANCY

Amasuko Mwingasalapo:

- SP (FANSIDAR)

- COARTEM

- QUININE

- IMBI

- NSHISHIBE

**41:41. PANSHITA YEFUMO IYI, MIKU INGA MWANWINE SP (FANSIDAR)? [NGA TABESHIBE, LEMBENI 88]. (ifipendwa)**

Data Field Name: FANSIDAR_TIMES

**42:42. NI INGA SP (FANSIDAR) MWAPELWE ELYO MWALI PABUKULU NA [ISHINA]? [NGATABESHIBE, LEMBENI 88]. (ifipendwa)**

Data Field Name: FANSIDAR_TABLETS_NUMBER

**43:43. [ICHIANDWA 9: UKUPEKANYA UKUPAPA] NOMBA NDEFWAYA UKWIPUSHA PALWA KUPPEKANYA UKUPAPA (Saleni)**

**44:44. BUSHE MWAFUMFWAPO AMASHIWA MUKUPEKENYA KWAKUPAPA MUUMYENSHI 6 YAPITA? [NGA IYO NANGULA TAMWISHIBE, YALATOLOKA YEKA] (ubwingi)**

Data Field Name: BIRTHPREPAREDNESS_MESSAGES

Amasuko Mwingasalapo:

- EMUKWAI

- IYO

- NSHISHIBE

**45:45. NINSHI ATEMWA NIBANI BAMIPELE ILYASHI ILI? [SALENI PAMASUKO AYALIPO] (ubwingi)**

Data Field Name: BIRTHPREPAREDNESS_MESSAGES_PROVIDER

Amasuko Mwingasalapo:

- BA SMAG (SAFE MOTHERHOOD ACTION GROUP)

- BA COMMUNITY HEALTH WORKER

- BA DOCTOR / BA NURSE / BA MIDWIFE

- PA RADIO

- PA TELEVISION

- BAMBI

**46:46. [ICHIANDWA 11: ABASAMBILILA UKUPAPASHISHA, INCHENDE YAKUPAPILAKO NA POSTNATAL CARE] NOMBA NDEFWAYA UKWIPUSHA PA KUPAPA KWA [ISHINA], NIKWI MWAPAPILE [ISHINA], NO KUCHECHETA [ISHINA] ICHIPATALA NAPANSHITA YAKUPAPA NO BUMI (Saleni)**

**47:47. NANI AMYAFWILE UKUPAPA KWA [ISHINA]? [SALENI AMASUKO YONSE ALIPO. MWIBELENGA AMASUKO. Fwailisheni] FIMBI? [FWAILISHENI PA MUNTU AWAFWILISHA ELYO MULEMBE UBWASUKO MWALAPELWA. NGA BAMWASUKA ATI TAPALI ABAYAFWILE, FWAILISHENI PAKUTI MUSANGE NGACHAKUTI ABAKALAMBA EKOBALI PANSHITA YAKUPAPA.] (ubwingi)**

Data Field Name: ASSISTED_DELIVERY_PERSON

Amasuko Mwingasalapo:

- BA DOCTOR

- BA NURSE

- BA CLINICAL OFFICER

- BA MIDWIFE

- BA EHT

- BA TRADITIONAL BIRTH ATTENDANT

- BA COMMUNITY HEALTH WORKER

- BAMBI BA HEALTH WORKER

- BA LUPWA ATEMWA ABANABO

- ABAKALMBA BAMBI

- BAMBI

- NANGU UMO

- NSHISHIBE

**48:48. MWAPAPILEKWI [ISHINA]? [SALENI PAMASUKO AYALIPO. FWAILISHENI IFISHINKA PALWA NTULO] (ubwingi)**

Data Field Name: PLACE_DELIVERY

Amasuko Mwingasalapo:

- PANG'ANDA YAKWANA CHIFYASHI

- PANG'ANDA IMBI

- KU GOVERNMENT HOSPITAL

- KU GOVERNMENT CLINIC/HEALTH CENTER

- KU GOVERNMENT HEALTH POST

- KU MISSION HOSPITAL / CLINIC

- KUMBI KU PUBLIC

- KU PRIVATE HOSPITAL

- KU PRIVATE CLINIC

- KU PRIVATE MATERNITY HOME

- KUMBI PRIVATE

- KUMBI

**49:49. [IYI YALASALA YEKA UKWAPAPILA. NGAMWAYASUKA UBWASUKO YASALA YEKA. MWICHITA NGACHIMO. KABIYENI PALIPUSHO LYAKONKAPO] (ubwingi)**

Data Field Name: VERIFICATION_FACILITY_DELIVERY

Amasuko Mwingasalapo:

- KUCHIPATALA KWAKUPAPILA

- TEKUCHIPATALA KWAKUPAPILA

**50:50. [LEMBENI EMUKWAI PA LIPUSHO ILI. TAMUFWILE UKWIPUSHA BANACHIFYASHI IFILI FYONSE. LEMBENIFYE EMUKWAI NOKUYA PALIPUSHO YAKONKAPO. YALATOLOKA IINE] (ubwingi)**

Data Field Name: FACILITY_SKIP1

Amasuko Mwingasalapo:

- EMUKWAI

- IYO

**51:51. NOMBA NDEFWAYA UKUMWIPUSHAKO AMEPUSHO PALWA NSHITA ELYO MWAPAPILE [ISHINA].**

**MWALANDA UKUTI MWAPAPILE MU [ISHINA YA CHIPATALA]. MWAIKELEKO INSHITA INGA ELYO MWAPAPILE? [SALENI PAMASUKO YONSE AYALIPO] (ubwingi)**

Data Field Name: LENGTH_FACILITY_STAY

Amasuko Mwingasalapo:

- LESS THAN 6 HOURS

- 6-11 HOURS

- 12-23 HOURS

- 1-2 DAYS

- 3 DAYS OR MORE

- NSHISHIBE

**52:52. NOMBA NDEFWAYA TULANDE PAFYA KONKELOPO ELYO MWAPAPILE NELYO MWAPAPILE MUCHIPATALA. BUSHE PALI UWAMIMWENE [ISHINA] UBUMI ELYO MWAPAPILE? [PALI UKUTOLOKA NGA MWAYASUKA EMUKWAI NANGULA IYO KULI ILI PUSHO] (ubwingi)**

Data Field Name: NEWBORN_PNC_POSTDISCHARGE

Amasuko Mwingasalapo:

- EMUKWAI

- IYO

**53:53. NDEFWA UKALANDA PAKUMO KWA [ISHINA] UBUMI ILYO MWAPAPA – NGA, UMUNTU ULEPIMA [ISHINA], UKUMONA UMUTOTO WAMWANA, NANGULA UKUMONA UMWANA NGA LIBWINO. ELYO [ISHINA] MWA PAPILE BUSHE KWALI UWAMIMWENEKO UBUMI BWA MWANA? [NGA IYO, YALATOLOKA YEKA] (ubwingi)**

Data Field Name : NEWBORN_PNC_NONFACILITY_NONASSISTED

Amasuko Mwingasalapo:

- EMUKWAI

- IYO

**54:54. BUSHE ICI CHACHITIKEFYE UMUKUMO, ATEMWA MIKUIYINGI? [NGA MIKU IYINGI, YALATOLOKA YEKA] (ubwingi)**

Data Field Name : NEWBORN_PNC_NONFACILITY_NONASSISTED_TIMES

Amasuko Mwingasalapo:

- UMUKUMO

- UKUPITILILA UMUKUMO

**55:55. NINSHITA NSHI YAPITILEPO ELYO MWAPAPILE PAKUCHITIKA FI? [SALENI PASUKO AYALIPO] (ubwingi)**

Data Field Name : NEWBORN_TIMING_FIRST_PNC_ONLY_1

Amasuko Mwingasalapo:

- LESS THAN 1 HOUR AFTER BIRTH

- 1-23 HOURS AFTER BIRTH

- 1-2 DAYS AFTER BIRTH

- 3-6 DAYS AFTER BIRTH

- 7 DAYS OR MORE AFTER BIRTH

**56:56. PANUMA YAKUPAPA PAPOSELE NSHITA INGA PAKUMICHECHETA UKU? [SALENI PAMASUKO AYALIPO] (ubwingi)**

Data Field Name : NEWBORN_TIMING_FIRST_PNC_MORETHAN_1

Amasuko Mwingasalapo:

- LESS THAN 1 HOUR AFTER BIRTH

- 1-23 HOURS AFTER BIRTH

- 1-2 DAYS AFTER BIRTH

- 3-6 DAYS AFTER BIRTH

- 7 OR MORE DAYS AFTER BIRTH

**57:57. BUSHE NANI AMICHECHETE [ISHINA] PA CHIPATALA PAKASHITA KALYA? [SALENI PAMASUKO AYALIPO] (ubwingi)**

Data Field Name : NEWBORN_PNC1_PERSON

Amasuko Mwingasalapo:

- BA DOCTOR

- BA NURSE

- BA CLINICAL OFFICER

- BA MIDWIFE

- BA EHT

- BA SMAG

-BA TRADITIONAL BIRTH ATTENDANT

- BA COMMUNITY HEALTH WORKER

- BAMBI BA HEALTH WORKER

- BA LUPWA NAGU ABANANDI

- BAMBI

**58:58. PAKUCHECHETA UKU, BUSHE IFI FYALICHITIKWE PALI [ISHINA]? [SALENI PAMASUKO AYALIPO. BELENGENI AMASUKO NO KULEMBA UBWASUKO BWABANYINEFWE]. (ubwingi)**

Data Field Name : NEWBORN_SIGNAL_FUNCTIONS_NONFACILITY_

Amasuko Mwingasalapo:

- EXAMINE CORD

- COUNSEL YOU ON DANGER SIGNS FOR NEWBORNS

- ASSESS THE TEMPERATURE OF YOUR BABY

- COUNSEL YOU ON BREASTFEEDING AND OBSERVE YOUR BABY BREASTFEEDING

- WEIGH THE BABY

- IDENTIFICATION AND REFERRAL OF ILLNESS

- EDUCATION ON HOME CARE FOR THE SICK INFANT

- EDUCATION ON HYGIENE, HAND WASHING AND SANITATION

**59:59. ILYO MWAPAPILE ABAMIPAPISHE BALISAYA, BUSHE KWALI UWAMICHECHETEKO UMBI PA BUMI BWENU? [IYI NAYIKWATA UKUTOLOKA YEKA] (ubwingi)**

Data Field Name : MATERNAL_PNC_POST_SBA

Amasuko Mwingasalapo:

- EMUKWAI

- IYO

**60:60. ELYO MWAPAPILE [ISHINA], BUSHE KWALI UWACHECHETE UBUMI BWENU? UMUNTU UWALEMO UBUMI BWENU, UWAPALA UKUMIPUSHA AMEPUSHO PALWA BUMI BWENU NOKU MICHECHETA. [IYI YATOLOKA YEKA]?**

**(ubwingi)**

Data Field Name : MATERNAL_PNC_NO_SBA

Amasuko Mwingasalapo:

- EMUKWAI

- IYO

**61:61. BUSHE UKU KUCHECHETA KWACHITIKE UMUKUMO NANGULA IINGI? (ubwingi)**

Data Field Name : MATERNAL_PNC_POST_SBA_TIMES

Amasuko Mwingasalapo:

- UMUKUMO

- UKUPITILILA UMUKUMO

**62:62. BUSHE PAPITILE NSHITA NSHI ELYO UKWISI KUMIMONA KWA CHITIKE? (ubwingi)**

Data Field Name : MATERNAL_TIMING_FIRST_PNC_ONLY_1

Amasuko Mwingasalapo:

- LESS THAN 1 HOUR AFTER BIRTH

- 1-23 HOURS AFTER BIRTH

- 1-2 DAYS AFTER BIRTH

- 3-6 DAYS AFTER BIRTH

- 7 DAYS OR MORE AFTER BIRTH

**63:63. BUSHE PAPITILE NSHITA NSHI NGA MWAPAPA ELYO MWAILE MUKUPAPA? (ubwingi)**

Data Field Name : MATERNAL_TIMING_FIRST_PNC_MORETHAN_1

Amasuko Mwingasalapo:

- LESS THAN 1 HOUR AFTER BIRTH

- 1-23 HOURS AFTER BIRTH

- 1-2 DAYS AFTER BIRTH

- 3-6 DAYS AFTER BIRTH

- 7 DAYS OR MORE AFTER BIRTH

**64:64. BUSHE NANI WAMICHECHETE PA BUMI PALI KALYA KA SHITA? (ubwingi)**

Data Field Name : MATERNAL_PNC1_PERSON

Amasuko Mwingasalapo:

- BA DOCTOR

- BA NURSE / BA CLINICAL OFFICER

- BA MIDWIFE

- BA EHT

- AMA SMAG

-BA TRADITIONAL BIRTH ATTENDANT

- BA COMMUNITY HEALTH WORKER

- BAMBI BA HEALTH WORKER

- BA LUPWA ATEMWA IFUBUSA

- ABAKALAMBA BAMBI

**65:65. [ICHIANDWA 13: UKUPIMISHA KWA HIV] NOMBA NDEFWAYA UKUMISHA AMEPUSHO PALWA KUPISHA KWA HIV NO KULANSHANYA (Saleni)**

**66:66. BUSHE BALIMIPELE HIV PANSHITA MWALIPABUKULU NA [ISHINA] NGA CHIMO PALWA ANTENATAL CARE NA [ISHINA]? [NGA IYO YATOLOKA IINE] (ubwingi)**

Data Field Name : OFFERED_HIV_TEST

Amasuko Mwingasalapo:

- EMUKWAI

- IYO

**67:67. NSHILEFWA UKWISHIBA IFYASANGWAMO, BUSHE BALIMIPIMA PALWA HIV NGA CHIMO PALWA ANTENATAL CARE NABA [ISHINA]? (ubwingi)**

Data Field Name : TOOK_HIV_TEST

Amasuko Mwingasalapo:

- EMUKWAI

- IYO

- NSHISHIBE

**68:68. NSHILEFWA UKWISHIBA IFYASANGWAMO, BUSHE MWAPOKELELA IFYATUMBWIKEMO MU HIV TEST NGA CHIMO PALWA ANTENATAL CARE? [NGA MWASALA IYO YALA TOLOKA IINE] (ubwingi)**

Data Field Name : RECEIVED_RESULTS_HIV_TEST

Amasuko Mwingasalapo:

- EMUKWAI

- IYO

- NSHISHIBE

**69:69. NSHILEYA UKWISHIBA IFYASANGWAMO, BUSHE MWALIKEBO PAMO NABO PALWA FYATUMBUKAMO MU HIV TEST NGA CHIMO PALWA ANTENATAL CARE? (ubwingi)**

Data Field Name : RECEIVED_RESULTS_COUNSELED_HIV_TEST

Amasuko Mwingasalapo:

- EMUKWAI

- IYO

- NSHISHIBE

**[TOTELENI BANA CIFYASHI PANSHITA BAKASHITA BASENDEMO ULUBALI] (Saleni)**
